# GBIS: the information system of the German Genebank

**DOI:** 10.1093/database/bav021

**Published:** 2015-05-07

**Authors:** Markus Oppermann, Stephan Weise, Claudia Dittmann, Helmut Knüpffer

**Affiliations:** Leibniz-Institut für Pflanzengenetik und Kulturpflanzenforschung (IPK) Gatersleben, OT Gatersleben, Corrensstraße 3, 06466 Stadt Seeland, Germany

## Abstract

The German Federal *ex situ* Genebank of Agricultural and Horticultural Crop Species is the largest collection of its kind in the countries of the European Union and amongst the 10 largest collections worldwide. Beside its enormous scientific value as a safeguard of plant biodiversity, the plant genetic resources maintained are also of high importance for breeders to provide new impulses. The complex processes of managing such a collection are supported by the Genebank Information System (GBIS). GBIS is an important source of information for researchers and plant breeders, e.g. for identifying appropriate germplasm for breeding purposes. In addition, the access to genebank material as a sovereign task is also of high interest to the general public. Moreover, GBIS acts as a data source for global information systems, such as the Global Biodiversity Information Facility (GBIF) or the European Search Catalogue for Plant Genetic Resources (EURISCO).

**Database URL:** http://gbis.ipk-gatersleben.de/

## Introduction

Currently, there are about 250 000 higher plant species in the world [conservative estimate, according to Ungricht (1)]. These include ∼7000 crop plant species [35 000 if ornamentals and forest plants are included (2)]. Crop plants are a major source for human and animal nutrition. Plants are cultivated by man also for fibre, oil, starch and other industrial purposes. Moreover, they play an important role as medicinal plants, for chemical and pharmaceutical industry and as renewable resources [e.g. (3, 4)]. Hence, it is indispensable to preserve plant genetic resources for future generations.

The collection, maintenance and characterization of crop plants and their wild relatives are important contributions towards the preservation of biological diversity. In particular, genebanks play an important role for the long-term conservation of plant genetic resources for food and agriculture (PGRFA). Around the world, there are about 1750 genebank collections (5). Therefore, about 625 collections are maintained in Europe comprising more than 2 million accessions (6). Their focus is not only on the aspect of pure conservation. The large genetic diversity stored in genebanks is also used to provide new impulses to plant breeding, e.g. by adding new alleles to existing breeding stocks (7).

The German Federal *ex situ* Genebank of Agricultural and Horticultural Crop Species maintained by IPK Gatersleben is amongst the 10 largest collections worldwide and the largest one in the countries of the European Union, totalling over 151 000 accessions. It has been continuously developed over the past 70 years [e.g. (8, 9)]. Beside the headquarters located in Gatersleben, there exist two further branch stations. Since 2002, the material from the Braunschweig Genetic Resources Centre (BGRC), the former West German genebank, was transferred to IPK to form the Federal Collection.

The genebank collection comprises 3220 plant species belonging to 768 genera (10). There are 31 genera with more than 1000 accessions each, but also 501 genera with less than 9 accessions each, including 199 genera represented by a single accession each. The genebank has material from 150 countries from all continents except Antarctica.

## The genebank information system of IPK

The present Genebank Information System (GBIS) has been developed since 2002 in the frame of the fusion of the collection of the former BGRC and the IPK genebank (11). It subsequently replaced earlier versions of the IPK genetic resources documentation system (12–15), as well as documentation systems of the BGRC collection (16) and the IPK branches (17, 18).

A genebank information system at IPK has to meet numerous and complex requirements.

The smallest unit kept separately in a genebank is called an accession, which is represented by physical objects such as seed samples, grow outs (plants from an accession grown together in a field plot) or lines (material preserved *in vitro* or *in cryo*). All these materializations of an accession share their origin and common information. Genebank accessions may enter the genebank collection in either of two ways: directly collected by a collector or an expedition, or provided by another collection or institution called ‘donor’.

The accessions are being preserved (i) as seed samples in cold storage facilities (ca. 96% of the collection), with the need for regular rejuvenation of the seed stock by growing in the field, (ii) vegetatively under field or greenhouse conditions, (iii) *in vitro* storage of plant tissues and (iv) cryopreservation in liquid nitrogen. Presently, about 8000 accessions are being rejuvenated per year; thus, the average storage duration of a seed accession is around 20 years (10). On request of users, i.e. plant breeders, researchers, but also private persons, the genebank is distributing research material amounting to over 30 000 samples in response to more than 1500 individual requests per year (10).

The highly complex work of the genebank maintenance is supported by the management component of the Genebank Information System (GBIS/M). Moreover, via the online search system GBIS/I (see below), information on genebank accessions is provided to external users around the world.

### Data domains and technical aspects

**Figure 1.**
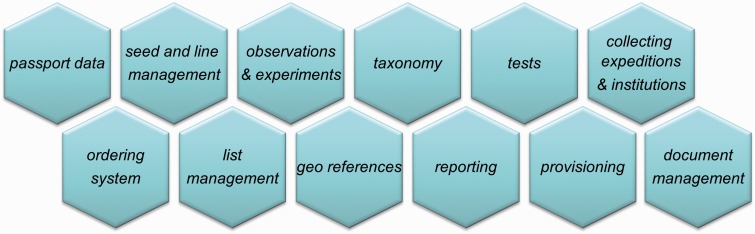
Data domains in GBIS.

Taking into account the different purposes of the Genebank Information System, the system structure can be categorized into data domains (Figure 1). The central object of the system is the accession in the sense of an anchor for the information belonging to the collection items. It is part of the passport data domain. In correspondence to the data domains, the implementation of the applications took place in different subprojects starting with a core system covering the basic functionalities.

To give an impression about the scope of GBIS, the most important functional domains of Figure 1 are briefly described below.

#### Passport data

Passport data includes basic information about the *where*, *when* and *what* of an accession, e.g. information about the collecting mission or the donor institute, collection site, names, numbers; complemented by data about the availability and other status information, different crop classifications, existence of specimens and images.

#### Seed and line management

Information necessary for the management of the genebank objects, such as storage location, availability of seed samples or lines of each accession, germinability of seeds or results of storage disease tests, generated and managed mainly in genebank workflows. Details of the workflow support are described in ‘Internal GBIS management applications GBIS/M and GBIS/B’.

#### Observations and experiments

An indispensable part of the work of genebanks is the phenotypic characterization of accessions (19). Phenotypic data is also called characterization and evaluation (C&E) data. Characterization refers to recording highly heritable traits, usually controlled by a single or very few genes, while evaluation refers to traits depending on several to many genes, whose expression is usually environment-dependent. From the point of view of database management, however, we do not distinguish between characterization and evaluation data. Phenotypic data is primarily obtained by observations during each regeneration cycle (20). Seed regeneration is usually driven by factors such as the availability of seeds or their germination capacity, rather than by statistical considerations. Thus, the available phenotypic data forms a non-orthogonal data set (21). Nevertheless, the analysis of this data allows meaningful results, such as the identification of promising new alleles (22) to support the selection of material for breeding and research programmes. The data model is designed to handle observations on heterogeneous accessions, i.e. populations consisting of different genotypes. There is a distinction between the definition of the traits with their different scales and the observations themselves that can be summarized in experiments. Traits can be organized in descriptors using a predefined crop-specific vocabulary derived from descriptor lists (23). However, this can only be guaranteed for data observed directly at IPK (characterization and primary evaluation data). Non-compliant data (secondary evaluation data) will be stored as accession-referenced files.

#### Taxonomy

Information about scientific names (present, but also historical determinations) of genebank accessions is fundamental. The taxonomic hierarchy, e.g. family, genus, species and infraspecific taxa, is reflected in the system.

#### Tests

Support for the just-in-time documentation of the results of health tests (e.g. for *in vitro* cultures) and germinability (of seed samples) on sample level.

#### Collecting expeditions and institutions

The plant material preserved in the genebank originates partly from collecting expeditions in many countries (largely conducted by, or in collaboration with IPK) or from donor institutions (e.g. research institutes, plant breeders, botanical gardens or other genebanks). This data allows for tracking the origin of an accession and provides access to further information about an accession.

#### Ordering system

To support the key task of genebanks, i.e. to provide plant (most often seed) samples to breeders, researchers and the public domain, comprehensive information needs to be managed. This includes, amongst others, the name and address of the person/institution requesting the material, the order (and the items ordered), the date of purchase and the status of the material to be delivered. Moreover, data which needs to be provided in accordance with the International Treaty (International Treaty on Plant Genetic Resources for Food and Agriculture, http://www.planttreaty.org/, as of 2014-11-28) are stored here.

#### List management

For many workflows, specific sets of accessions, samples or grow outs have to be defined, based on various criteria. Examples are: lists of accessions that need to be sown for regeneration, or lists of seed samples to be fetched out from the cold storage for distribution. Therefore, a very flexible query builder was implemented for list management and reporting. As a special feature, lists of objects can be fixed, irrespectively of changes in the underlying data.

#### Document management

To assist the user in managing all kinds of documents that refer to an accession. These can be herbarium specimens, as well as photos, scanned file cards or fieldbook pages and other digital documents.

Except for the geo references, the remaining domains belong to common functionalities such as reporting for querying and statistics, and provisioning of internal and external user accounts.

### GBIS applications

**Figure 2.**
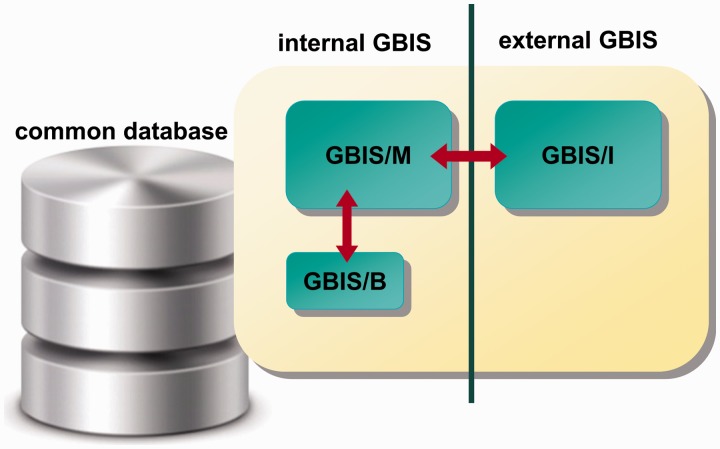
Overview of the GBIS architecture.

GBIS consists of three subsystems: (i) a desktop application for supporting the internal collection management (**GBIS/M**anagement), (ii) an application for mobile data collection in the field (**GBIS/B**onitur, German for scoring or (field) observations on plants) and (iii) an Internet search and order portal for external genebank users (**GBIS/I**nformation, **i**nternet). The three applications share a common database (Figure 2).

#### Internal GBIS management applications GBIS/M and GBIS/B

**Figure 3.**
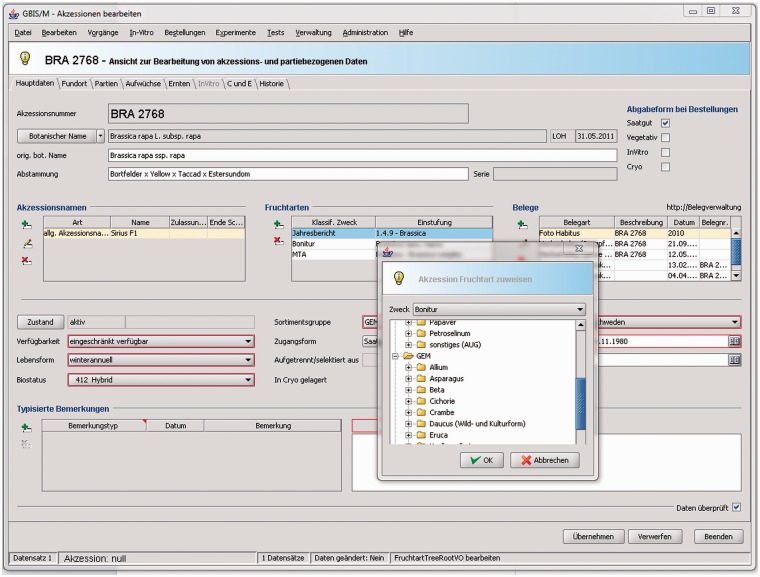
Form with main data of an accession. It is part of the management module and enables access to all information about an accession. It allows the curators to manage all this information at a central location. Details of the domain objects related to the accession can be edited on the different tabs.

The first subsystem, GBIS/M (Figure 3), forms the internal management component of GBIS. It can be described as a two-faced tool that (i) has been designed to support a wide range of workflows within the genebank (Figure 5) and also (ii) to provide internal users with access to the GBIS database for curating genebank data outside a workflow context. All data domains described in ‘Data domains and technical aspects’ are reflected by the modules of this application (Figure 1).

##### Accession management

Accessions are materialized, e.g. as seed samples, lines or grow-outs (plants grown together in the field). The accession management part of GBIS/M provides an accession-oriented access with the possibility of viewing and editing data. For every accession, it is possible to trace histories of numbers and other identifiers, names, botanical determinations and origins as well as phenotypic data. Furthermore, preservation management data is accessible here.

##### Work flow support

**Figure 4.**
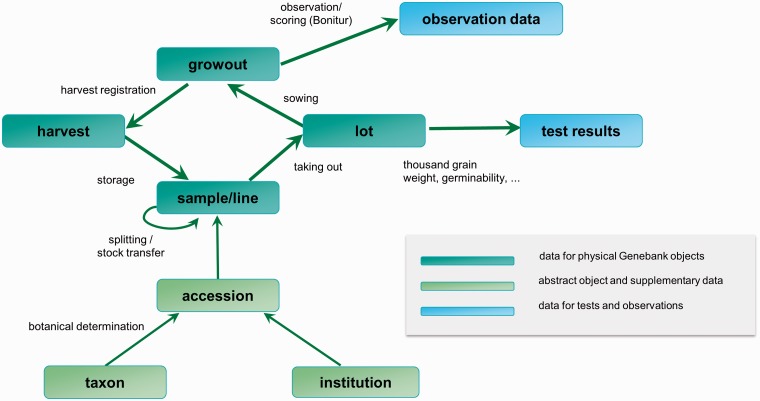
Important domain objects and data management-related genebank workflows.

GBIS/M is highly integrated in the daily work routine of the genebank staff. For every supported workflow, the data are preprocessed to give an optimized view of information to match the needs of the task. In Figure 4, the core objects of the system and the information flow during some workflows between these objects are shown.

**Figure 5.**
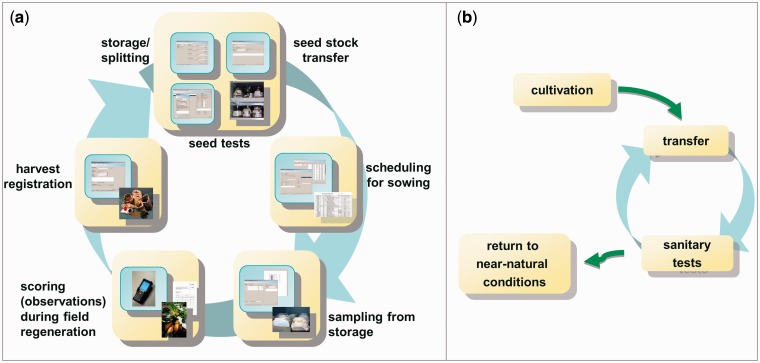
Typical management workflows of the genebank, which are supported by GBIS. (**a**) shows the ‘seed cycle’ for seed-propagated material, whereas (**b**) shows the life cycle of plant material stored *in vitro.*

This strong integration of GBIS/M in the working processes of the genebank leads, in case of seed and line management, to a closed system (Figure 5). Each step is based on the previous one and can benefit from the information generated there.

The second subsystem, GBIS/B, is a Java application that runs on mobile devices equipped with a barcode scanner (24). It supports the user in recording phenotypic data of field trials. For this, the relevant data (crop, traits to be observed, and observations from previous grow-outs of the same accession) is transferred from GBIS to a mobile device. After registering the observations in the field, the data are synchronized with the GBIS database. The whole surrounding workflow is fully integrated in the GBIS/M subsystem.

#### External GBIS: Internet search and order portal GBIS/I

**Figure 6.**
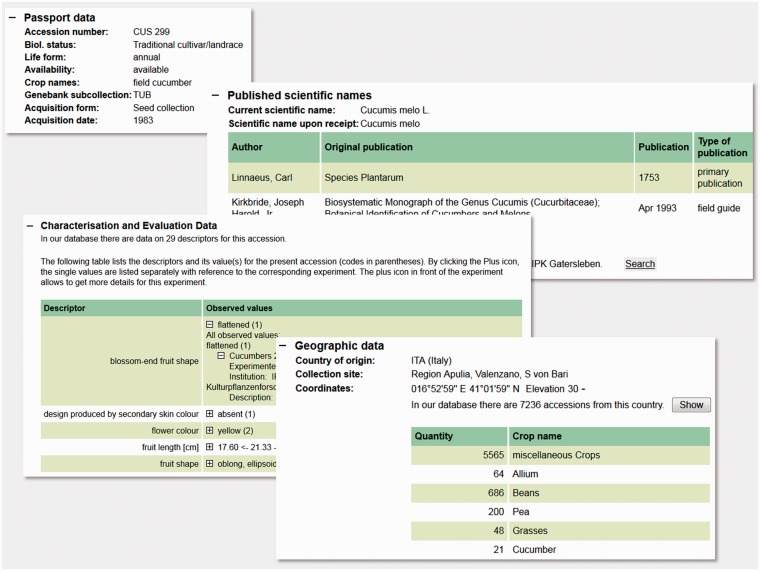
Compilation of screenshots of the accession details view in the GBIS/I application. It shows a kaleidoscope of data from different data domains supporting scientists, breeders and other interested users to select appropriate genebank accessions. It represents the view for external users onto the data managed by the genebank curators as shown in Figure 3.

For external users, especially plant breeders and researchers, the web portal GBIS/I is the main source of information about genebank material. It allows for searching and filtering accessions by various well-defined criteria and their combinations in a simple stacked way or by defining complex Boolean concatenation. For certain characteristics, a free-text search is implemented. If a crop category is chosen, also phenotypic data become searchable. The search results are first shown in an overview table where selected accessions can be put in a wish list for ordering or data export. By clicking on an accession number, the user can view all available details of this accession. Moreover, GBIS/I offers a shopping-cart function enabling registered users to request material of interest online. It also fulfils the requirements of the International Treaty on Plant Genetic Resources for Food and Agriculture by implementing a click-wrap-mechanism for signing Standard Material Transfer Agreements (SMTA). The system is featured with an order tracking function. All data shown is refreshed at least daily, based on the real-time data in the common database. A compilation of screenshots displaying accession detail information is shown in Figure 6.

### Implementation

The database schema is realized on an Oracle 11g relational database management system (Oracle Corporation, http://www.oracle.com/, as of 2014-10-13) in a top-down-design based on the object model developed during the requirement analysis phase (2002–2006). The comprehensiveness results in a highly normalized database schema comprising 111 domain-specific tables as well as 78 look-up tables for decoding. To speed-up data retrieval, materialized views are used. As a sustainability-by-design feature, we keep the schema readable and understandable by non-informaticians or external IT specialists.

The implementation is characterized by a strong customization for the needs of the German genebank and IPK’s IT infrastructure, which is dominated by Oracle technologies. Both the desktop application GBIS/M and the web application GBIS/I make use of the Oracle Application Development Framework (http://www.oracle.com/technetwork/developer-tools/adf/, as of 2014-10-14) (25). GBIS/I runs on a Oracle WebLogic Server (http://www.oracle.com/us/products/middleware/cloud-app-foundation/weblogic/, as of 2014-10-14). Due to the use of commercial software, the GBIS source code is not deposited in an open source repository but can be made available on request.

**Table 1:** Origin of accessions in IPK’s genebank by continents.

Continent	Accessions	Countries	Most frequent countries
Africa	12 139	35	Ethiopia (7462), Morocco (1731), Libya (1086)
Americas	13 883	25	USA (3887), Peru (3130), Mexico (1402)
Asia	23 734	40	Turkey (5020), Iran (3521), China (2384)
Europe	75 136	49^a^	Germany (20634), Italy (7264), former Soviet Union (4209)
Oceania	507	2	Australia (413), New Zealand (94)
Unknown	25 470		

^a^Including countries not existing any longer.

## Outlook

In its present state, GBIS is already an important source of information for various external systems, such as the European search catalogue for plant genetic resources, EURISCO (European Search Catalogue for Plant Genetic Resources of the European Cooperative Programme for Plant Genetic Resources, http://eurisco.ecpgr.org/, as of 2014-10-14) (26), the Global Biodiversity Information Facility, GBIF (http://www.gbif.org/, as of 2014-10-14) (27), Mansfeld’s World Database of Agricultural and Horticultural Crops (http://mansfeld.ipk-gatersleben.de/, as of 2014-10-14) (28), the Garlic and Shallot Core Collection (GSCC) (29), or the Life Science search engine LAILAPS (30). Access to GBIS data is provided in several ways, e.g. using the Biocase PyWrapper (31), as file export or as direct database link. GBIS supports different data exchange standards, such as the Multi-Crop Passport Descriptors (FAO/Bioversity: Multi-Crop Passport Descriptors V.2 (MCPD V.2) (2012), http://www.bioversityinternational.org/e-library/publications/detail/faobioversity-multi-crop-passport-descriptors-v2-mcpd-v2/, as of 2015-02-02) and Access to Biological Collections Data (ABCD 2003: Content definition: the ABCD Schema. CODATA/TDWG Task Group on Access to Biological Collection Data. http://www.bgbm.org/TDWG/CODATA/Schema/, as of 2015-02-02). However, in order to increase the attractiveness for research and breeding, genebank information systems such as GBIS need to be more closely connected with data from beyond the classical plant genetic resources domain. This includes information from the—omics areas, such as genotyping, metabolomics and proteomics. In the future, such information will help improving the collection management in terms of identifying duplicates, ensuring genetic purity or establishing core collections. Moreover, breeders will benefit from a better identification of appropriate germplasm for breeding purposes. This applies in particular to the enhancement of breeding lines with regard to phenotypic traits, such as drought tolerance. The added value that could be created by systematically using genebank material, cannot be estimated highly enough.

As a proof of concept, IPK is currently preparing the genotyping of the whole barley collection (∼20 000 accessions). It is planned to connect the information gained from this initiative with GBIS.

With the experience from the current subsystems, a next generation of the system, GBIS2, is under development. It transforms the rich-client architecture into a state-of-the-art web-based system, to benefit from the use of the same technology for all subsystems. This gives a chance to keep it open for further enhancements by a leaner development process. Restrictions of the current technology will be overcome and the subsystems can be built in a more scalable way.
